# Self-reported history of intensity of smoking is associated with risk factors for suicide among high school students

**DOI:** 10.1371/journal.pone.0251099

**Published:** 2021-05-13

**Authors:** Meenakshi Dasagi, Dale S. Mantey, Melissa B. Harrell, Anna V. Wilkinson

**Affiliations:** UTHealth, School of Public Health, Austin, TX, United States of America; University of California San Diego School of Medicine, UNITED STATES

## Abstract

**Objective:**

To examine the relationship between current cigarette smoking patterns and three established risk factors for suicide using nationally representative data of high school students in the United States.

**Methods:**

We analyzed cross-sectional data from the national Youth Risk Behavior Surveillance Survey (YRBSS)–United States, 2017. Multivariable, logistic regressions examined the association between 3 cigarette smoking behaviors [i.e., past 30-day cigarette (n = 13,731), frequent (n = 1,093) and heavy (n = 880) smoking] and 3 risk factors for suicidal outcomes [feeling sad or hopeless, suicidal ideation, suicide plan] assessed over the previous year.

**Results:**

Among high school cigarette smokers, smoking 11 or more cigarettes per day (i.e., heavy smoking) was associated with 3.43 (95% CI: 1.69, 6.94) greater odds of reporting feeling sad or hopeless, 2.97 (95% CI: 1.60, 5.51) greater odds of reporting suicidal ideations, and 2.11 (95% CI: 1.34, 3.32) greater odds of reporting having ever planned a suicide attempt, controlling for covariates.

**Conclusions:**

Our study shows that it is not simply cigarette smoking, but heavy cigarette smoking that is a risk factor for suicidal outcomes among adolescents.

**Public health implications:**

A comprehensive plan is needed to accommodate heavy adolescent smokers who are at increased suicidal risk.

## Introduction

Suicide is the second leading cause of death among individuals aged 10–19 years old [1]. Between 2000 and 2017 deaths from suicide surpassed deaths from homicide among older adolescents aged 15 to 19 years old [1]. Risk factors for suicide during adolescence include feelings of hopelessness (i.e., depression), suicidal ideation (i.e., thoughts of suicide), and planning a suicide attempt [2]. National data reveal that the prevalence of each of these risk factors is increasing among adolescents in the United States: feelings of hopelessness increased from 26.1% in 2009 to 31.5% in 2017, suicidal ideation increased from 14.5% in 2007 to 17.2% in 2017 and planning a suicide attempt increased from 10.9% to 13.6% in 2009 to 2017 [3]. It is crucial that we develop evidence-based practices to mitigate these risk factors.

Previous research has found a consistent association between cigarette smoking and risk factors for suicide among adolescents [4, 5]. A recent systematic review found that current cigarette smokers (i.e., smoked at least 100 cigarettes and smoked in the past 30 days) were nearly twice as likely to report suicidal ideation, suicide planning, and suicide attempts compared to non-smokers [6]. Moreover, cigarette smoking is associated with depression [7], a leading predictor of suicidal behavior [8].

While there is an established link between current cigarette smoking and risk factors for suicide, no studies to date have examined whether the intensity and frequency of smoking are related, too. Expanding our understanding of the relationship between smoking intensity/frequency and risk factors for suicide may provide public health practitioners with critically needed information on elevated risk for suicidal behaviors. Thus, by addressing this gap in the literature, using nationally representative data, our study may improve suicide prevention interventions.

## Methods

### Study sample

In this cross-sectional study, we examined data from the national Youth Risk Behavior Surveillance Survey (YRBSS)–United States, 2017. The national YRBSS is a federally funded classroom-based paper and pencil survey conducted every two years on odd years [9]. The national YRBSS uses a three-stage, cluster sample design to obtain a nationally representative sample of students in grades 9 through 12 [3]. The target population consists of all public and private school students in grades 9 through 12 in all 50 states and the District of Columbia [3]. For the 2017 national YRBSS, 14,956 questionnaires were completed in 144 private and public schools. However, 191 questionnaires were excluded due to quality issues in the data, resulting in 14,765 usable questionnaires and thus a sample size of 14,765 [3]. According to the YRBSS User guide [9], the school response rate was calculated as 144 of the 192 sampled schools that participated (144/192 = 75%), while the student response rate was calculated as 14,765 of the 18,324 sampled students that submitted questionnaires (14,765/18,324 = 81%). Finally, the overall response rate was calculated as school response rate multiplied by student response rate, (75%*81% = 60%) [9].

#### Analytic sample sizes

Overall, n = 1,034 (7%) of the YRBSS sample (n = 14,765) had missing data on socio-demographic variables and were excluded from all analyses resulting in a total sample of n = 13,731 participants. Additionally, we examined two subsamples derived from past 30-day cigarette smokers (n = 1,223).

The first subsample was used to explore frequency of cigarette smoking. A total of n = 130 cigarette smokers had missing data on frequency of cigarette smoking, resulting in a subsample of n = 1,093 past 30-day cigarette smokers for the analysis of frequent cigarette smoking. The second subsample was used to explore heavy cigarette smoking. A total of n = 343 cigarette smokers had missing data on heavy cigarette smoking, resulting in a subsample of n = 880 past 30-day cigarette smokers for the analysis of heavy cigarette smoking.

**Fig 1:**
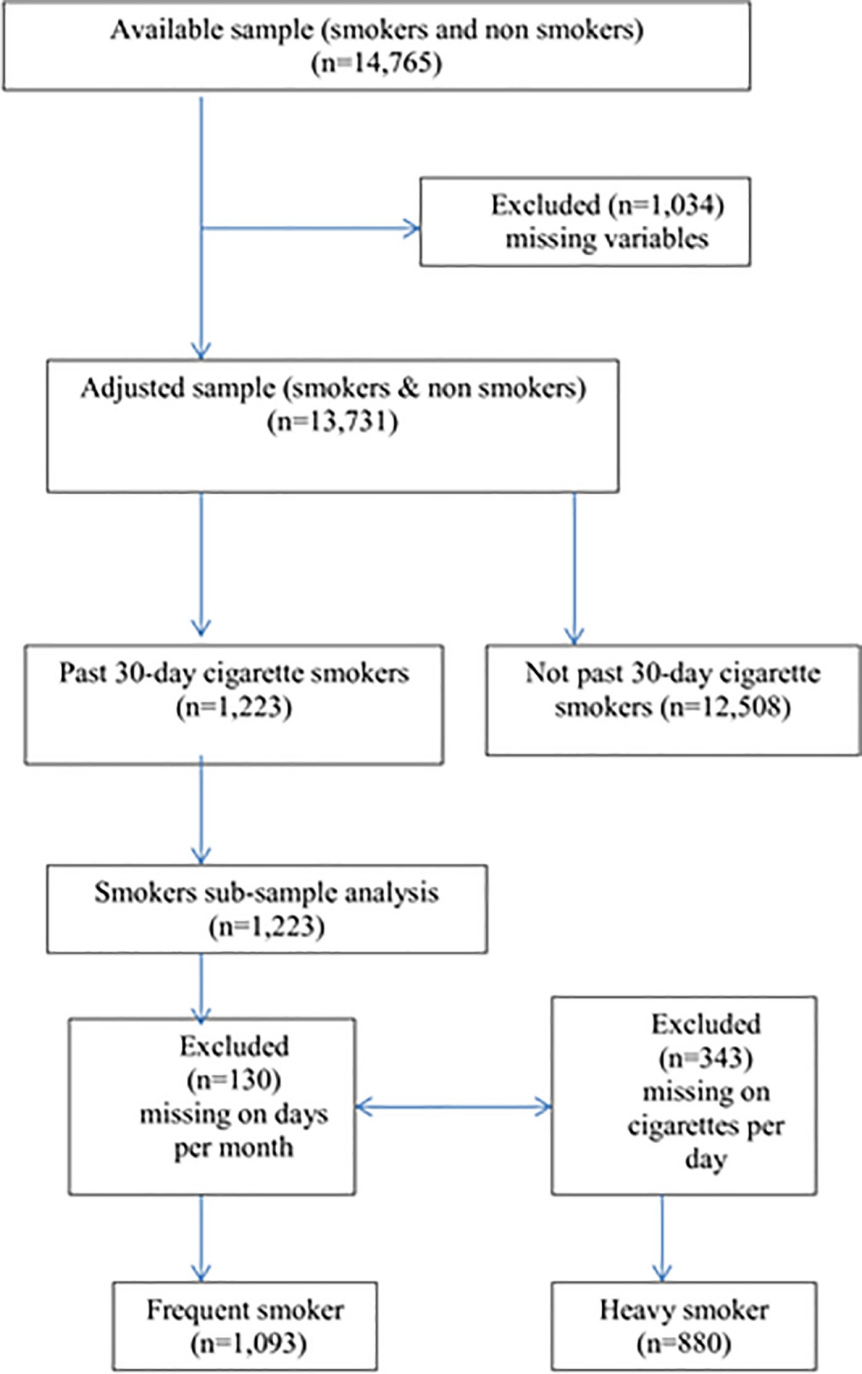
Sample selection.

Of note, the study samples of frequent cigarette users and heavy cigarette users are not mutually exclusive. Here from among the sample size of n = 880, 7.3%, or n = 65 of the high school students smoked more than 10 cigarettes in a day and reported smoking on more than 20 days out of the past 30 (Fig 1).

### Measures

#### Independent variables

*Current cigarette smoking*. The first independent variable we examined was self-reported history of current cigarette smoking. Each participant was asked, “During the past 30 days, on how many days did you smoke cigarettes?” Individuals who reported smoking cigarettes between 1 to 30 days were defined as current cigarette smokers. Those who reported 0 days were coded as non-current users and served as the referent group.

*Frequent cigarette smoking*. The second independent variable was frequent cigarette smoking. Participants who reported smoking 20 to 30 days out of the past 30 days were considered frequent cigarette smokers, per CDC guidelines [3]. Participants who reported smoking 1 to 19 days served as the referent group.

*Heavy cigarette smoking*. The third independent variable was intensity of cigarette smoking and was based on the self-reported number of cigarettes smoked daily. Each participant was asked, “During the past 30 days, on the days you smoked, how many cigarettes did you smoke per day?” Participants who reported smoking 11 or more cigarettes per day were categorized as heavy smokers, per CDC guidelines [3]. Participants who smoked 1 to 10 cigarettes per day served as the referent group.

#### Dependent variables

This study examined three risk factors for suicidal outcomes. Participants were asked to self-report experiencing any of the following within the past twelve months: “did you ever feel so sad or hopeless almost every day for two weeks or more in a row that you stopped doing some usual activities?” (i.e., feeling sad or hopeless), “did you ever seriously consider attempting suicide (i.e., suicidal ideation) and “did you make a plan about how you would attempt suicide” (i.e., suicide plans). Possible responses for all three of these questions were dichotomous: “no” (referent outcome) and “yes.”

#### Covariates

Socio-demographic covariates included race/ethnicity, sex, and grade. Race/ethnicity was categorized into the four groups: non-Hispanic white (referent), Hispanic/Latino, non-Hispanic Black, and non-Hispanic other. For the purposes of this study “non-Hispanic other” included: Asian, non-Hispanic Multiracial, American Indian or Alaska Native (AIAN)/ Native Hawaiian or other Pacific Islander (NHOPI). Sex was a dichotomous variable; males served as the referent group. Grade was an ordinal variable with groups being 12^th^ grade (referent), 11^th^ grade, 10^th^ grade and 9^th^ grade.

### Statistical analysis

Data were weighted to be representative of 9^th^ through 12^th^ grade (i.e., high school) students attending public and private schools in the United States. The study hypotheses were tested using multiple logistic regression in separate models to assess the relationship between each cigarette smoking behavior and each of the study outcomes. First, three multiple logistic regressions examined the relationship between past 30-day (i.e., current) cigarette smoking and feeling sad/hopeless, suicidal ideations, and planning a suicide attempt in the past 12-months. Next, three multiple logistic regressions examined the relationship between frequent cigarette smoking (i.e., 20 or more days per month) and feeling sad/hopeless, suicidal ideation, and planning a suicide attempt in the past 12-months. And last, three multiple logistic regressions examined the relationship between heavy cigarette smoking (i.e., 11 or more cigarettes per day) and feeling sad/hopeless, suicidal ideation, and planning a suicide attempt in the past 12-months. All regression analyses controlled for sex, grade and race/ethnicity.

## Results

### Descriptive statistics

Table 1 presents the results from the bivariate analyses of the three suicide risk measures. Self-reported history of feeling sad or hopeless was greater among females (41%) than males (20.8%; p<0.001), as well as self-reported history of suicidal ideations (females = 22.0% and males = 11.6%; p<0.001). Additionally, significantly more females (17.0%) reported having a suicide plan than males (9.3%; p<0.001).

**Table 1:** Demographic characteristics and tobacco use behaviors by risk for suicide among high school students (Youth Risk Behavior Surveillance Survey, 2017).

			Risk for Suicide	
	Total^a^ (n = 13,731)	Sad or Hopeless^b^ (n = 13,731)	Suicidal Ideation^c^ (n = 13,731)	Suicide Plan^d^ (n = 13,731)
**Demographic Characteristics**				
**Sex**		**P<0.0001**	**P<0.0001**	**P<0.0001**
Male	49.3%	20.8%	11.6%	9.3%
Female	50.7%	41.0%	22.0%	17.0%
**Grade**		P = 0.2669	P = 0.7055	P = 0.5510
12^th^ Grade	23.1%	30.0%	17.0%	13.0%
11^th^ Grade	23.9%	32.0%	17.2%	14.0%
10^th^ Grade	25.7%	32.0%	17.2%	14.0%
9^th^ Grade	27.3%	30.0%	16.0%	12.4%
**Race/Ethnicity**		**P = 0.0174**	**P = 0.0030**	**P = 0.0082**
NHW^**e**^	53.5%	30.0%	17.1%	12.5%
Hispanic/Latino	22.9%	33.3%	16.1%	13.1%
NHB^**f**^	13.4%	28.1%	13.9%	12.1%
NHO^**g**^	10.3%	34.7%	20.6%	18.0%
**Cigarette Use Behaviors**				
**Current**^**h**^		**P<0.0001**	**P<0.0001**	**P<0.0001**
No	91.2%	29.0%	15.2%	11.9%
Yes	8.8%	51.2%	34.1%	26.8%
**Frequent**^**i**^		P = 0.4450	P = 0.1491	P = 0.2589
	(n = 1,093)	(n = 1,093)	(n = 1,093)	(n = 1,093)
1–19 days	70.0%	50.3%	31.7%	25.3%
20–30 days	30.0%	53.3%	39.7%	30.2%
**Heavy**		**P = 0.0026**	**P = 0.0019**	**P = 0.0055**
	(n = 880)	(n = 880)	(n = 880)	(n = 880)
1–10 per day	90.3%	49.7%	31.8%	25.8%
10+ per day	9.7%	73.7%	53.9%	38.4%

a. Total adjusted sample size

b. Self-reported feeling “so sad or hopeless almost every day for two weeks or more in a row that you stopped doing some usual activities” in the past year

c. Self-reported having seriously considered attempting suicide in the past year

d. Self-reported having “made a plan” to commit suicide in the past year

e. NHW = non-Hispanic white

f. NHB = non-Hispanic black

g. NHO = non-Hispanic other; includes “Asian, non-Hispanic,” “American Indian/Alaska Native, non-Hispanic,” or “native Hawaiian and other Pacific Islanders, non-Hispanic”

h. Self- reported as having smoked a cigarette in the past 30-days

i. Self-reported number of days smoked in the past 30days

j. Self-reported number of cigarettes smoked per day

As seen in Table 1, bivariate analyses revealed statistical differences in cigarette use behavior by study outcomes. Among the full adjusted sample (n = 13,731), current cigarette smokers reported greater prevalence of feeling sad or hopeless (p<0.001), suicidal ideation (p<0.001) and suicide plan (p<0.001) compared to non-smokers. Among current cigarette smokers, those who reported heavy smoking had a greater prevalence of feeling sad or hopeless (p = 0.003), suicidal ideation (p<0.002) and suicide plan (p = 0.006) compared to those who were not heavy smokers. Of note, the prevalence of suicide risk was not statistically different between frequent cigarette smokers and non-frequent cigarette smokers.

### Smoking behaviors and suicidal risk

As seen in Table 2, past 30-day cigarette smokers had greater odds of feeling sad or hopeless (AOR: 3.00; 95% CI: 2.36, 3.80), reporting suicidal ideation (AOR: 3.15; 95% CI: 2.55, 3.90) and reporting having ever planned a suicide attempt (AOR: 3.04; 95% CI: 2.34, 3.92), controlling for covariates.

**Table 2:** Association between current cigarette use and risk for suicide among high school students (n = 13,731).

		Risk for Suicide	
	Sad or Hopeless^a^ AOR^d^ (95% CI)	Suicidal Ideation^b^ AOR (95% CI)	Suicide Plan^c^ AOR (95% CI)
**Current Cigarette Smoking**^e^			
No	1.00	1.00	1.00
Yes^e^	3.00***(2.36, 3.80)	3.15***(2.55, 3.90)	3.04***(2.34, 3.92)
**Sex**			
Male	1.00	1.00	1.00
Female	2.73***(2.33, 3.20)	2.20***(1.84, 2.63)	2.05***(1.73, 2.44)
**Grade**			
12^th^ Grade	1.00	1.00	1.00
11^th^ Grade	1.13 (0.97, 1.32)	1.08 (0.86, 1.36)	1.19 (0.92, 1.54)
10^th^ Grade	1.15 (0.99, 1.32)	1.11 (0.91, 1.34)	1.19 (0.91, 1.56)
9^th^ Grade	1.05 (0.91, 1.22)	1.05 (0.88, 1.25)	1.11 (0.91, 1.35)
**Race/Ethnicity**			
Non-Hispanic White	1.00	1.00	1.00
Hispanic/Latino	1.28**(1.07, 1.53)	1.00 (0.83, 1.21)	1.14 (0.90, 1.45)
Non-Hispanic Black	1.01 (0.82, 1.25)	0.87 (0.73, 1.03)	1.08 (0.81, 1.45)
Non-Hispanic Other^f^	1.34*(1.07, 1.70)	1.36**(1.08, 1.71)	1.66***(1.32, 2.10)

*p < .05

**p < .01

*** p < .001

a. Self-reported feeling “so sad or hopeless almost every day for two weeks or more in a row that you stopped doing some usual activities” in the past year

b. Self-reported having seriously considered attempting suicide in the past year

c. Self-reported having “made a plan” to commit suicide in the past year

d. AOR = Adjusted Odds Ratio, adjusted for sex, grade, and race/ethnicity

e. Self- reported as having smoked a cigarette in the past 30-days

f. “Non-Hispanic Other” includes “Asian, non-Hispanic,” “American Indian/Alaska Native, non-Hispanic,” or “native Hawaiian and other Pacific Islanders, non-Hispanic.”

As seen in Table 3, frequent cigarette smoking (i.e., 20 to 30 out of the past 30 days) was not associated with increased risk for feeling sad or hopeless, suicidal ideation, or having ever planned a suicide attempt, after controlling for covariates.

**Table 3:** Association between frequent cigarette use and risk for suicide among high school students (n = 1,093).

		Risk for Suicide	
	Sad or Hopeless^a^ AOR^d^ (95% CI)	Suicidal Ideation^b^ AOR (95% CI)	Suicide Plan^c^ AOR (95% CI)
**Frequent Cigarette Smoking**^e^			
1 to 19 days	1.00	1.00	1.00
20 to 30 days	1.09 (0.78, 1.51)	1.38 (0.83, 2.30)	1.30 (0.82, 2.06)
**Sex**			
Male	1.00	1.00	1.00
Female	2.69***(1.95, 3.72)	1.99***(1.41, 2.81)	2.03***(1.46, 2.84)
**Grade**			
12^th^ Grade	1.00	1.00	1.00
11^th^ Grade	0.86 (0.51, 1.46)	0.89 (0.55, 1.42)	1.25 (0.88, 1.78)
10^th^ Grade	0.98 (0.65, 1.49)	0.91 (0.54, 1.51)	1.25 (0.79, 1.97)
9^th^ Grade	0.86 (0.49, 1.52)	1.17 (0.74, 1.84)	2.02**(1.22, 3.34)
**Race/Ethnicity**			
Non-Hispanic White	1.00	1.00	1.0
Hispanic/Latino	1.56 (0.99, 2.46)	1.15 (0.68, 1.95)	1.15 (0.60, 2.21)
Non-Hispanic Black	0.62 (0.26, 1.45)	0.88 (0.38, 2.06)	0.54 (0.21, 1.37)
Non-Hispanic Other^f^	2.26**(1.35, 3.80)	1.12 (0.68, 1.84)	1.54 (0.78, 3.06)

*p < .05

**p < .01

*** p < .001

a. Self-reported feeling “so sad or hopeless almost every day for two weeks or more in a row that you stopped doing some usual activities” in the past year

b. Self-reported having seriously considered attempting suicide in the past year

c. Self-reported having “made a plan” to commit suicide in the past year

d. AOR = Adjusted Odds Ratio, adjusted for sex, grade, and race/ethnicity

e. Self-reported number of days smoked in the past 30 days

f. “Non-Hispanic Other” includes “Asian, non-Hispanic,” “American Indian/Alaska Native, non-Hispanic,” or “native Hawaiian and other Pacific Islanders, non-Hispanic”

As seen in Table 4, heavy cigarette smoking (i.e., 11 or more cigarettes per day) was associated with greater odds of reporting feeling sad or hopeless (AOR: 3.43; 95% CI: 1.69, 6.94), reporting suicidal ideation (AOR: 2.97; 95% CI: 1.60, 5.51), and reporting having ever planned a suicide attempt (AOR: 2.11; 95% CI: 1.34, 3.32), controlling for covariates.

**Table 4:** Association between heavy cigarette use and risk for suicide among high school students (n = 880).

		Risk for Suicide	
	Sad or Hopeless^a^ AOR^d^ (95% CI)	Suicidal Ideation^b^ AOR (95% CI)	Suicide Plan^c^ AOR (95% CI)
**Heavy Cigarette Smoking**^e^			
1 to 10 / day	1.00	1.00	1.00
11+ / day	3.43**(1.69, 6.94)	2.97***(1.60, 5.51)	2.11*(1.34, 3.32)
**Sex**			
Male	1.00	1.00	1.00
Female	2.96***(2.10, 4.15)	2.27*(1.63, 3.16)	2.16***(1.51, 3.10)
**Grade**			
12^th^ Grade	1.00	1.00	1.00
11^th^ Grade	0.92 (0.54, 1.59)	0.92 (0.55, 1.55)	1.30 (0.92, 1.85)
10^th^ Grade	1.03 (0.67, 1.58)	0.81 (0.45, 1.47)	1.24 (0.79, 1.94)
9^th^ Grade	0.86 (0.48, 1.55)	1.15 (0.73, 1.82)	2.06**(1.22, 3.46)
**Race/Ethnicity**			
Non-Hispanic White	1.00	1.00	1.0
Hispanic/Latino	1.58 (1.00, 2.49)	1.12 (0.65, 1.92)	1.15 (0.58, 2.28)
Non-Hispanic Black	0.72 (0.30, 1.74)	0.65 (0.35, 1.19)	0.60 (0.22, 1.64)
Non-Hispanic Other	2.17*(1.21, 3.88)	1.01 (0.58, 1.78)	1.46 (0.68, 3.15)

*p < .05

**p < .01

*** p < .001

a. Self-reported feeling “so sad or hopeless almost every day for two weeks or more in a row that you stopped doing some usual activities” in the past year

b. Self-reported having seriously considered attempting suicide in the past year

c. Self-reported having “made a plan” to commit suicide in the past year

d. AOR = Adjusted Odds Ratio, adjusted for sex, grade, and race/ethnicity

e. Self-reported number of cigarettes smoked per day in the past 30 days

f. “Non-Hispanic Other” includes “Asian, non-Hispanic,” “American Indian/Alaska Native, non-Hispanic,” or “native Hawaiian and other Pacific Islanders, non-Hispanic”

## Discussion

Our findings suggest that heavy cigarette smoking (e.g., ≥ half pack of cigarettes per day) is related to suicide risk among adolescents. Our results are consistent with prior research demonstrating a link between current cigarette smoking and risk factors for suicide [6], and extend prior research by observing that heavy, but not frequent, smoking also is linked to increased suicide risk among adolescents [6]. Further study is needed to determine if, and to what degree, heavy smoking may increase the risk of suicide by affecting nicotinic pathways in the brain that increases the risk of suicide [6, 10]. Our results have implications for developing interventions targeted at adolescents as they identify those adolescents who are at increased suicide risk based on their smoking patterns.

Exposure to nicotine during adolescence influences the development of the prefrontal cortex including the development of inhibitory capacities [11, 12]. Poor inhibitory control is linked with greater dependence on nicotine [13], underscoring the possibility that development process may be disrupted among heavy tobacco dependent adolescent smokers. In previous studies, smoking intensity has been linked to psychological distress among adolescents [14]. Among adolescents, psychological distress can manifest as externalizing behaviors, such as smoking [14]. In turn externalizing behaviors, including smoking, are associated with social disinhibition [15] and poor inhibitory control [16, 17] and increased risk for suicidal behavior [18]. Future research should examine relationships between other tobacco product use (e.g., e-cigarettes) that can deliver increased intensity of nicotine and suicidal risk [19, 20].

Our study builds on prior literature as it reveals that it is heavy cigarette smoking that is associated with reduced mental wellbeing: adolescent heavy smokers are at increased risk for suicide relative to their peers who smoke fewer cigarettes per day. Further study is needed that explores the clinical implications and underlying mechanisms of this relationship. This includes looking into groups that maybe impacted by this relationship, such as those at elevated risk for both cigarette smoking and suicide during adolescence. Therefore, early and tailored preventive measures for this risk group is essential. Targeting impulse control, which is associated with various disruptive behavior disorders [21] and with smoking initiation [16], may serve to improve the efficacy of the interventions. In addition, longitudinal studies are needed to understand how the relationships between tobacco product use, impulse control, and suicide risk, change over time.

When smoking is initiated at a young age the risk of heavy smoking increases, which leads to increased nicotine dependence [22]. Given the negative impacts of nicotine exposure on adolescent brain development [11], heavy smoking may serve as a risk factor to identify adolescents particularly at high risk for suicide. These immediate adverse effect from nicotine addiction should not be underestimated [23].

Like all, our study had limitations. First, because we used a cross-sectional study design and smoking was assessed over the past 30 days, while feelings of sadness and hopeless, suicide ideation, and suicide plan were assessed over the past year, we cannot make causal inferences about the direction of the association between tobacco use behaviors and the suicidal risk factors. Second, the self-reported data on sensitive topics may have resulted in underreporting cigarette smoking and / or suicidal risk factors, due to social desirability influences. Third, the school response rate of 75% and student response rate of 81% resulting in an overall response rate of 60% [9], might have introduced selection bias to the results. Finally, it was not possible to control for potential confounders, such as substance use disorders [24] and impulsivity [13, 18, 21], that may affect tobacco smoking behaviors and suicidal risk factors.

Despite these limitations, the present study has the following strengths. It used a large nationwide representative sample of adolescents in the United States, which enhances the generalizability of the results. Our study differentiates between heavy and frequent smoking behaviors as potential risk factors for suicidal risk in adolescents. To the best of our knowledge, this is the first study that implicates heavy tobacco use in the risk for suicide among older teens compared to frequent smoking.

## Conclusions

As we frame a public health strategy, we need to consider that there are varied smoking behaviors among adolescents. Hence a comprehensive plan is needed to accommodate these different smoking behavior patterns. Heavy smokers are at increased suicidal risk. Though, further research is needed to understand the relationship observed in this study, these findings suggest a need for expanding education and increased suicidal prevention strategies for heavy adolescent tobacco smokers.
